# No Effect on Tumorigenesis in MG63 Cells Induced by Co-Cultured Mesenchymal Stem Cells

**DOI:** 10.1155/2022/4202439

**Published:** 2022-07-06

**Authors:** Xin Xing, Yue-Hua Hu, Yan Wang, Yi Shao, Min Zou

**Affiliations:** ^1^Department of Orthopaedics Surgery, The Third Hospital of Hebei Medical University, Shijiazhuang 050000, China; ^2^Basic Medical College, Hebei Medical University, Shijiazhuang 050017, China; ^3^Obstetrics and Gynecology Department, North China University of Science and Technology Affiliated Hospital, Tangshan 063000, China; ^4^Orthopaedic Institute of Hebei Province, Shijiazhuang 050000, China; ^5^Key Laboratory of Biomechanics of Hebei Province, Shijiazhuang 050000, China

## Abstract

Osteosarcoma is a kind of bone tumor with an extremely high malignant degree and often occurs in adolescents. Mesenchymal stem cells are believed to play an important role in the microenvironment of osteosarcoma, but whether they promote or inhibit the development of osteosarcoma is controversial. In this study, the coexpression of mesenchymal stem cells (MSCs) with osteosarcoma cell MG63 was used to explore the effect of MSCs on MG63. We found that co-culture of MSCs with MG63 did not affect the proliferation, invasion, and migration of MG63 cells, nor did it significantly affect the epithelial- and glial-mesenchymal transformation of MG63 cells. Therefore, in this study, we obtained a new concept that MSCs neither promote nor inhibit the occurrence and development of osteosarcoma.

## 1. Introduction

Osteosarcoma (OS) is a malignant bone tumor that tends to occur in adolescents [1]. It is highly malignant and often complicated by irregular bone growth and distant metastases to the lungs and liver [2]. Despite advances in treatment, the prognosis for patients with OS has not improved over the past two decades. In the absence of metastasis, the 5-year survival rate of OS patients is 65%, but 20% in the patients with metastasis, and the survival time of patients with lung metastasis is significantly reduced [2].

Studies have shown that the tumor microenvironment (TME) and bone microenvironment play a crucial role in the development and metastasis of osteosarcoma [3]. Paget believes that the tumor microenvironment can provide fertile soil for tumor cells to seed and promote tumor growth and development [4]. The microenvironment of osteosarcoma consists of bone cells, stromal cells, vascular cells, and immune cells. Among them, bone cells include osteoblasts, osteoclasts, and osteocytes, and stromal cells include mesenchymal stem cells (MSCs) and fibroblasts. Communication between osteosarcoma and the bone microenvironment is mediated by a variety of cytokines and soluble growth factors [5]. A study published in 2016 showed that MSCs can enable osteosarcoma cells to acquire proliferation-promoting antiapoptotic abilities. The study showed that compared with the control group, the proliferation of osteosarcoma cells in MSCs conditioned medium was significantly accelerated and the ability to resist apoptosis was significantly enhanced [6]. Other studies have shown that MSCs can promote the growth and migration of osteosarcoma cells by releasing vesicles [6, 7]. Interleukins, monocyte chemotactic protein-1 (MCP-1), and chemokines released by MSCs in the tumor microenvironment promote tumor migration and invasion [8]. A large number of literature studies have indicated that MSCs can act as sensors between tumor cells and the microenvironment and they can be involved in immune suppression, stimulation of angiogenesis, inhibition of apoptosis of cancer cells, induction of epithelial-mesenchymal transformation, and other links to promote tumor growth [9–11], but some studies have shown that it can inhibit tumor growth by inhibiting angiogenesis, AKT signaling, and Wnt signaling pathway; but its role in the occurrence and development of osteosarcoma is still unknown [12, 13].

MSCs are important sources of stem cell therapy in regenerative medicine. Distributed in various tissues of the body, bone marrow mesenchymal stem cells (BMSCs) have shown good therapeutic effects in various degenerative diseases in animal models and human clinical trials [14]. In addition, MSCs have a good targeted transport effect and can be used as vectors to target tumor growth sites and perform effective clinical functions [15]. In order to further determine whether MSCs can be used as a carrier for the treatment of OS, it is necessary to confirm whether MSCs can promote or inhibit the occurrence and development of OS. Therefore, we co-cultured MSCs with MG63 and tested relevant indicators to determine the exact role of MSCs in the growth of osteosarcoma.

## 2. Materials and Methods

### 2.1. Co-Culture of MSC and MG63

MG63 cell lines were from Procell Life Science and Technology Co. Ltd. (CL-0157). The isolation and purification of MSCs were described in the literature [2]. Conventional culture in 6 cm^2^ dishes with DMEM medium (10569010, Thermo Fisher Scientific, USA) supplemented with 10% fetal bovine serum in a 37°C incubator. Trypsin (15400054, Thermo Fisher Scientific, USA) was used for cell passage, then MSC and MG63 were co-cultured at a 24 mm transwell with 0.4 *μ*m pore polycarbonate membrane insert (3412, Corning, USA), and MSC was placed in the upper layer and MG63 in the lower layer. Protein and RNA were extracted after MSC and MG63 were co-cultured for 24 h at co-culture rations of 1 : 1 and 1 : 2.

### 2.2. CCK-8

CCK-8 kit (C0038, Beyotime, China) was used to detect the proliferation of MG63 cells in the control group and co-culture group. Firstly, the number of cells was determined. In the control group, 100 *μ*L MG63 cells were added into the lower layer of 96-well TRANSWELL, and the number of MG63 cells was 2000; in the co-culture group, 2000 MG63 cells were added into the lower layer, 2000 MSCs cells were added into the upper layer, and the total medium was 200 *μ*l. After co-culture for 24 hours, the culture dishes of the upper MSCs cells were taken out. Then, CCK-8 solution was added into two 96-well plates, respectively, and incubated in the cell culture box for 1 h. Finally, the absorbance of the two groups of cells was detected by an enzyme plate to determine the number of cells.

### 2.3. Real-Time RT-PCR

MSCs and MG63 were co-cultured in different proportions, and RNA was extracted from two cells by trizol (15596026, Invitrogen) at a specified time. The purity and concentration were measured by NanoDrop2000. Reverse transcription (ZR102, ZOMANBIO, China) was performed according to company recommendations. RT-PCR reactions consisted of 10 *μ*l 2 × SYBR qPCR Mix (ZF102, ZOMANBIO, China), 1 *µ*L of both 10 *µ*M forward and reverse primers, 2 *µ*L of cDNA, and 17 *µ*L of H_2_O. The primer sequence details are shown in Table 1. The results were calculated via the 2^−△△CT^ method.

### 2.4. Western Blot

After different proportions of MSC and MG63 were co-cultured to a predetermined time, the supernatant of the upper and lower chambers of the 6-well cell culture plate was aspirated, and cells were cleaned twice with PBS. A protein extraction kit (SD-001, Invent Biotechnologies, Inc, China) was used for protein extraction. Lysis buffer was added and spilled on the ice for 5 min. Then, the protein lysate was filtered through a centrifugal column and placed into a centrifuge for 10 minutes under the condition of 10000 rpm and 4°C, and the clear fluid in the collection tube is the protein. NanoDrop 2000 was used to measure protein concentration. After that, the protein was prepared with a 5x loading buffer in proportion, and the sample was performed using publishing protocols [16, 17] with antibodies directed against *α*-smooth muscle actin (*α*-SMA, ab5694, Abcam, Cambridge, UK), E-cadherin (ab212059, Abcam), N-cadherin (AF4039, Affinity), Slug (ab51772, Abcam), Snail (ab216347, Abcam), and Vimentin (ab92547, Abcam); the dilution of these antibodies was 1 : 1000, and *β*-actin (ab8227, Abcam) and GAPDH (ab9485, Abcam) ratio of these two antibodies was 1 : 100000.

### 2.5. Cell Migration Assay

In the control group, MG63 were seeded in a 6-well plate alone. In the co-culture group, MG63 were seeded in a lower layer of the 6-transwell plate, and MSCs were seeded in the upper layer of the 6-transwell plate in different proportions. Then, a scratch was made through the cell layer using a sterile micropipette tip, and in the co-culture group, the scratch was just made in the lower layer. Pictures of cell scratches were taken under a microscope at 0 h and 24 h after scribing. The cell migration ability was evaluated by measuring the change of scratch space distance. The data were analyzed with Image-Pro Plus 6.0.

### 2.6. Invasion Assay

The well plate was coated with 100 ml 5% matrix adhesive and placed in the incubator for 2 h. After blank culture for 12 h, MG63 was digested and suspended again, and the number of cells was determined; 150 ml cell suspension containing 80,000 cells was added to each chamber lined with matrix adhesive. In the control group, blank minimum essential medium (MEM) was used in the upper layer, and MEM containing 20% serum was used in the lower layer. In the experimental group, the upper layer was cultured with the blank medium of MSCs, and the lower layer was also cultured with MEM containing 20% serum. After 24 h of culture, the medium was discarded, the upper compartment was fixed in methanol solution for 10 min, and then stained in crystal violet (V5265, sigma) solution for 10 min. The cells on the membrane of the compartment were erased, and the cells under the membrane were left for observation under the microscope.

### 2.7. Immunofluorescence

Immunofluorescence staining was performed using published protocols with antibodies directed against E-ca/*α*-SMA at a concentration of 1 : 200 [18].

## 3. Results

### 3.1. MSCs Had No Effect on Proliferation of MG63 Cells

**Figure 1:**
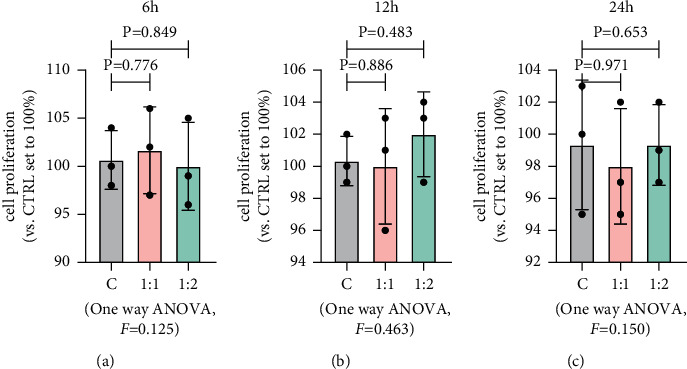
MSCs had no effect on proliferation of MG63. (a) MSCs and MG63 were co-cultured for 6 h at different culture ratios. Data are presented as the mean ± SD. *n* = 3 per group. (b) MSCs and MG63 were co-cultured for 12 h at different culture ratios. Data are presented as the mean ± SD. *n* = 3 per group. (c) MSCs and MG63 were co-cultured for 24 h at different culture ratios. Data are presented as the mean ± SD. *n* = 3 per group.

**Figure 2:**
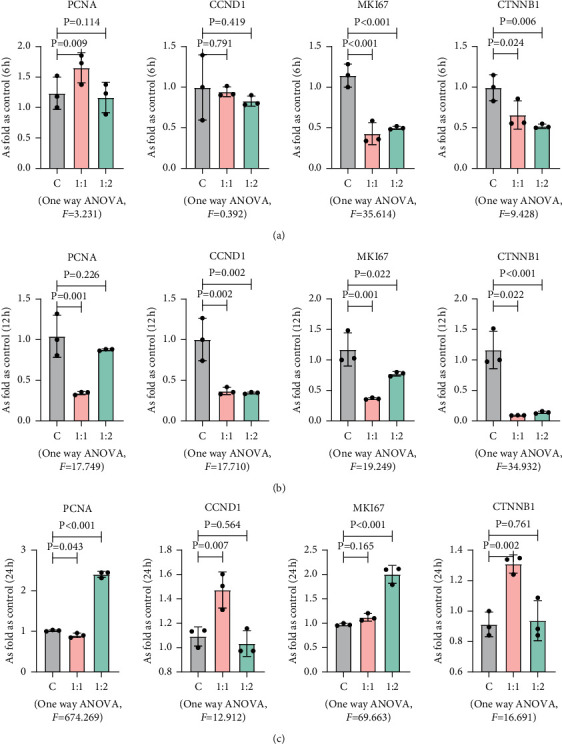
MSCs had no effect on proliferation-related genes of MG63. The mRNA levels of PCNA, CCND1, MKI67, and CTNNB1 in MG63 co-cultured with MSCs in different ratios at different times, measured by qRT-PCR. Data are presented as the mean ± SD. *n* = 3 per group. (a) MG63 and MSCs co-cultured in 1 : 0, 1 : 1, and 1 : 2 at 6 h. (b) MG63 and MSCs co-cultured in 1 : 0, 1 : 1, and 1 : 2 at 12 h. (c) MG63 and MSCs co-cultured in 1 : 0, 1 : 1, and 1 : 2 at 24 h.

In order to test whether MSCs can promote tumor cell proliferation, we performed CCK-8 assay and detected proliferation-related indicators by PCR. The results of CCK-8 showed that MSCs had no effect on the proliferation of MG63. Then, we examined the changes of related gene measurements at 6 h (Figure 1(a)), 12 h (Figure 1(b)), and 24 h (Figure 1(c)) after co-culture. However, contrary to CCK-8 results, the results of qRT-PCR have shown that the expression levels of PCNA, cyclin *D*, Ki67, and *β*-catenin were different. After 6 h of co-culture, compared with the MG63 group, the differences of proliferating cell nuclear antigen (PCNA), cyclin D1 (CCND1), and catenin beta 1 (CTNNB1) in the two co-culture groups were not statistically significant (*p* > 0.05), while the expression of marker of proliferation Ki67 (MKI67) in the two co-culture groups was significantly downregulated compared with the MG63 group, which was statistically significant (*p* < 0.05) (Figure 2(a)). After 12 h of co-culture, the expressions of PCNA and MKI67 in the group that cultured MSCs and MG63 at 1 : 2 and the expressions of CTNNB1 in the group where MSCs and MG63 were cultured in accordance with 1 : 1 had no statistical significance compared with that in the MG63 group (*p* > 0.05), while the rest had statistical significance (*p* < 0.05) (Figure 2(b)). After 24 h of co-culture, CCND1 and CTNNB1 expressions in the MSCs and MG63 group cultured in accordance with 1 : 2 and MKI67 group cultured in accordance with 1 : 1 were not statistically significant compared with those of the MG63 group (*p* > 0.05) (Figure 2(c)), while the rest were statistically significant (*p* < 0.05), and the specific reasons still need to be further explored.

### 3.2. MSCs Had No Effect on the Migration and Invasion of MG63

**Figure 3:**
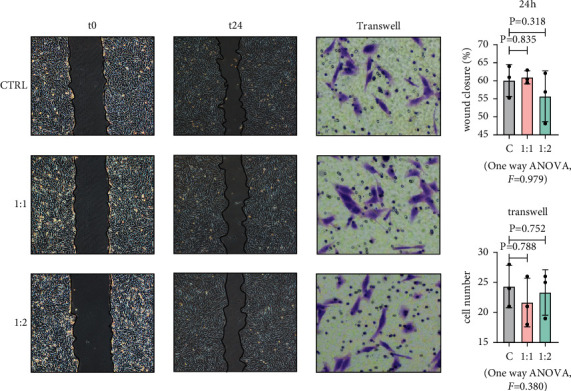
MSCs had no effect on migration and invasion of MG63. Migration experiment and invasion experiment of MSCs and MG63 in different co-culture ratios at 24 h. Bar = 50 *μ*m. Data are presented as the mean ± SD. *n* = 3 per group.

One of the most important factors in promoting the recurrence and growth of osteosarcoma is its aggressiveness. Therefore, for exploring the effect of MSCs on the migration and invasion function of MG63. We performed scratch and invasion tests on the control group and co-culture group. At first, we detect the migration function, the results show that the MSCs did not enhance or suppress the MG63 motility (Figure 3). Next, the impact of MSCs on the invasion ability of MG63 was evaluated by analyzing the invasion characteristics of osteosarcoma cells. We used transwell dishes to detect the invasion ability of MG63, the cells of the control group were cultured in a normal medium, and the culture medium for cultured MSCs was collected and cultured for the other group of cells. The number of MG63 in the lower layer can be observed (Figure 3). The results showed that MSCs had no effect on the invasion of MG63.

### 3.3. MSCs Did Not Affect the Activation of MG63 Epithelial-Mesenchymal Transition (EMT)

**Figure 4:**
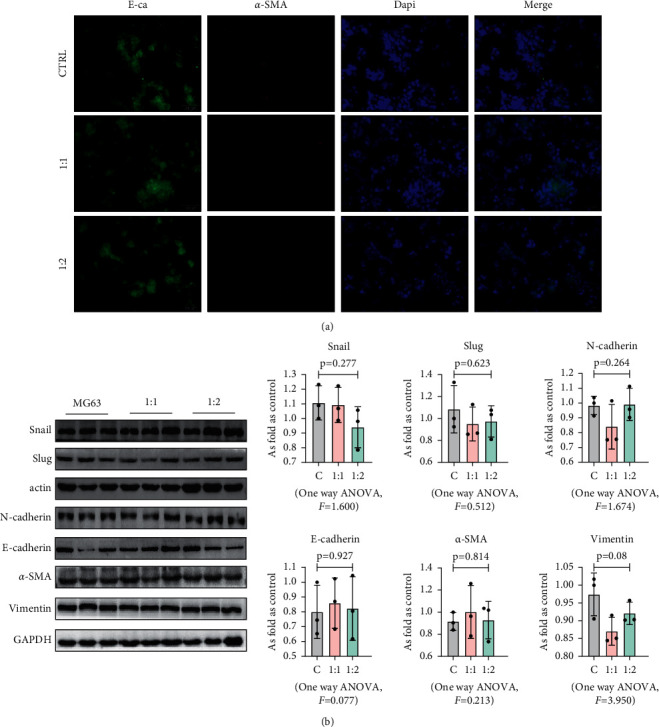
MSCs had no effect on the EMT of MG63. (a) Co-expression of E-cadherin and *α*-SMA in MG63 cells was observed by IF staining. Bar = 50 *μ*m. (b) Levels of Snail, Slug, N-cadherin, E-cadherin, *α*-SMA, and vimentin in MG63 cells, measured by western blotting. Data are presented as the mean ± SD. *n* = 3 per group.

**Figure 5:**
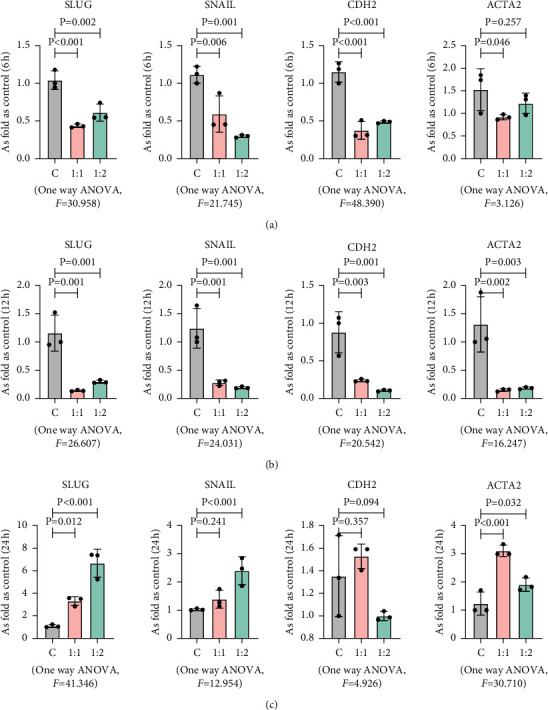
The co-culture of MSCs and MG63 had no effect on EMT-related genes. The mRNA levels of Snail, Slug, N-cadherin, and *α*-SMA in MG63 co-cultured with MSCs in different ratios at different times, measured by qRT-PCR. Data are presented as the mean ± SD. *n* = 3 per group. (a) MG63 and MSCs co-cultured in 1 : 0, 1 : 1, and 1 : 2 at 6 h. (b) MG63 and MSCs co-cultured in 1 : 0, 1 : 1, and 1 : 2 at 12 h. (c) MG63 and MSCs co-cultured in 1 : 0, 1 : 1, and 1 : 2 at 24 h.

EMT plays an important role in promoting tumor progression and metastasis. It has been shown that osteosarcoma cells that undergo EMT acquire invasive and metastatic characteristics [19]. Therefore, we investigated whether MSCs could induce EMT of MG63 by evaluating the main regulators in the EMT process, such as Snail, Slug, N-cadherin, E-cadherin, *α*-SMA, and vimentin, which were used for immunofluorescence double standard, and the results showed that there was no significant difference in the expression of *α*-SMA and E-cadherin at 24 h with different co-culture ratios (Figure 4). The results of western blot (Figure 4) and qRT-PCR (Figure 5) showed that compared with MG63 alone, the protein expression levels and gene expression levels of these indicators in MG63 cells co-cultured with MSC showed no significant changes (*p* < 0.05).

### 3.4. Effect of MSCs on MG63 Cytokine Release

**Figure 6:**
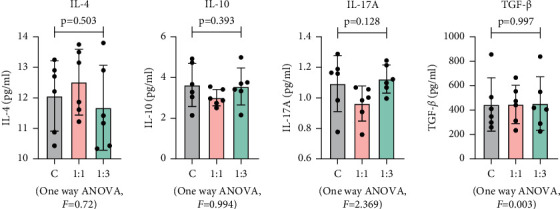
MSCs had no effect on cytokine release of MSCs. The concentration levels of IL-4, IL-10, IL-17A, and TGF-*β* in the medium were measured by ELISA. Data are presented as the mean ± SD. *n* = 6 per group.

In the TME, the effect of MSCs on tumor cells is largely dependent on the cytokines secreted by MSCs. Studies have shown that these cytokines can stimulate tumor cells leading to tumor development and metastasis by promoting migration and immune escape [20, 21]. In osteosarcoma cells, cytokine release is considered as a biomarker of tumor progression [22, 23]. To investigate the effects of MSCs on MG63 secretome composition, the levels of secreted cytokines were analyzed in two groups. The results have shown that there are no significant differences between the control group and the co-culture group in IL-4, IL-10, IL-17A, and TGF-*β* (Figure 6).

## 4. Discussion

Osteosarcoma is a primary tumor with high recurrence and metastasis, and these characteristics lead to poor prognosis [24]. The standard treatment for OS is surgery, chemotherapy, and radiation [25]. With these treatments, the rate of recurrence and metastasis of OS is still higher than 30%, and the 5-year survival of patients with recurrence and metastasis is lower than 25% due to the resistance of chemotherapy drugs [26]. Therefore, it is imperative to explore the broader pathogenesis of OS and thus explore new therapeutic approaches.

MSCs in OS microenvironment are BMSCs; in humans, they are adult pluripotent stem cells. BMSCs exist in the whole life cycle and play an important role in the maintenance and repair of tissue homeostasis [27]. As MSCs exist in a large number in the OS microenvironment and have their unique therapeutic potential and physiological functions, the related mechanisms of tumor microenvironment on the development of osteosarcoma have been extensively studied. However, as an important part of the tumor microenvironment, the role of MSCs on OS has not been determined yet. Therefore, in this study, we simulated the interaction between OS cells and MSCs in vivo, co-cultured MG63 with MSCs, and explored the real effect of MSCs on OS cells.

Many studies have expressed different views on the role of MSCs in OS. Some studies suggest that MSCs promote the occurrence and development of OS. Firstly, MSCs are thought to be the source of OS [28], and deletion of TP53 and Rb genes is a common cause of the transition from MSCs to OS cells [29]. Other studies have shown that MSCs can promote the invasion and metastasis of osteosarcoma cells. In in vivo experiments in rats, tumor formation and growth rates were higher in COS1NR (rat OS cells) alone subcutaneously compared with COS1NR co-implanted with rat MSCs in the first five weeks. MSCs were then injected intravenously at weeks 3 and 5 after COS1NR cells were inoculated subcutaneously. Although there was no significant difference in subcutaneous tumor growth, the number of pulmonary nodules in the MSCS injection group increased significantly compared with those without MSC injection [30]. In addition, gene expression profiles of MSC were also analyzed, and the results showed that some genes related to adhesion spots, cytokine receptors, and extracellular matrix receptor pathways were highly expressed in MSC, suggesting that MSC may participate in the occurrence and development of OS and promote its invasion and migration through these pathways [30]. It has also been reported that MSCs interact with OS cells, and OS cells also affect their microenvironment, causing MSCs to undergo metabolic reprogramming and secrete more lactic acid, thus further promoting the metastasis of OS [31]. These are different from our results, our results suggest that mesenchymal stem cells did not affect the proliferation and apoptosis of osteosarcoma cells nor did they affect their migration and invasion ability when OS cells were co-cultured with MSCs. As for the difference between the results of in vitro and reported in vivo experiments, we still need to explore further whether there are other factors influencing the microenvironment and the effect of MSCs on OS cells in vivo.

At the same time, in vivo experimental results in other articles supported that MSCs did not promote the growth and reproduction of OS. It has been suggested that although MSC is the source of a portion of OS cells, OS cells derived from MSC do not have tumor properties and are highly similar to normal MSCs [32], and it was also shown that MSC-derived OS cells extracted from OS mice did not induce tumorigenesis when injected into immunodeficient mice [32]. PI3K/AKT signal can be abnormally high expressed in OS cells [33], and the activation of this signal can promote the growth, proliferation, and adhesion of cells [34]. Correlation analysis of primary OS patients showed that the PI3K/AKT signal transduction is significantly highly correlated with the poor prognosis of OS [35]. Interestingly, in Cassibo's sarcoma, MSC can play an important antitumor role through exposure to AKT-dependent inhibitory activity [12]. Different tumors may have consistent effects. Therefore, MSCs may also inhibit the occurrence and development of osteosarcoma cells by inhibiting AKT signaling, but this needs to be further verified. Related studies on glioma diseases have found that MSCs have the ability to track tumor cells. MSCs were isolated from healthy people and labeled with fluorescence and then injected into the carotid artery of intracranial mice carrying human glioma, and MSCs were found in brain tumors. In addition, the survival rate of mice injected with MSCs was significantly higher than that of glioma mice not injected with MSCs [36]. Therefore, whether MSCs can be injected in vitro to inhibit the progression of osteosarcoma in some ways is our next research focus.

In conclusion, there are few studies on the effect of MSCs on osteosarcoma cell growth. The interaction and communication among MSCs, OS cells, TME, and stromal cells are extremely complex and play a decisive role in the progression or treatment of OS. Therefore, an in-depth study of the role and mechanism of MSCs on OS can provide a better clinical treatment direction for OS.

## 5. Conclusions

At present, the application of MSCs in tumor therapy is attracting more and more attention. However, in OS cells, the role of MSCs has shown different results in different studies, mainly manifested as the promotion and inhibition of MSCs on OS cells. MSCs affect tumor cells mainly through cytokines and chemokines, stromal cells, and the tumor microenvironment. In order to explore the specific effects of MSCs on OS and the possibility of MSCs treating OS, in this study, we used in vivo experiments to co-culture MCSs and MG63 and detected the effects of MSCs on MG63 apoptosis, proliferation, migration, and invasion ability, EMT progression signal. The results showed that the co-culture of MSCs and MG63 had no significant effect on the related functions of MG63. It is inconsistent with the previous result that MSCs can promote the occurrence and development of OS. At present, there are two main directions for the treatment of OS by MSCs. One is to activate or inhibit a certain target signaling pathway by the secretion regulation of MSCs and secreting related cytokines to restrict tumor growth. The second is to use MSCs as a carrier to achieve targeted tumor therapy. In this study, it was found that MSCs alone had no significant effect on OS through in vitro experiments, but its therapeutic effect on osteosarcoma as a carrier remains to be explored. Meanwhile, during the evolution of OS, MSCs from different sources will be recruited into TME, and the interaction between these MSCs and tumor cells remains unclear and needs further study. In general, MSCs have a broad application prospect in the clinical treatment of tumors, and more experiments are needed to study the molecular mechanism of its action on tumor cells.

## Figures and Tables

**Table 1:** Primers used for RT-PCR.

Gene	Forward	Reverse
PCNA	CAAGTAATGTCGATAAAGAGGAGG	GTGTCACCGTTGAAGAGAGTGG
CCND1	TCTACACCGACAACTCCATCCG	TCTGGCATTTTGGAGAGGAAGTG
MKI67	GAAAGAGTGGCAACCTGCCTTC	GCACCAAGTTTTACTACATCTGCC
CTNNB1	CACAAGCAGAGTGCTGAAGGTG	GATTCCTGAGAGTCCAAAGACAG
Slug	ATCTGCGGCAAGGCGTTTTCCA	GAGCCCTCAGATTTGACCTGTC
Snail	TGCCCTCAAGATGCACATCCGA	GGGACAGGAGAAGGGCTTCTC
CDH2	CCTCCAGAGTTTACTGCCATGAC	GTAGGATCTCCGCCACTGATTC
ACTA2	CTATGCCTCTGGACGCACAACT	CAGATCCAGACGCATGATGGCA

## Data Availability

Data are available upon request.

## References

[1] Mirabello Lh., Troisi R. J., Savage S. A. (2009). Osteosarcoma incidence and survival rates from 1973 to 2004: data from the surveillance, epidemiology, and end results program. *Cancer*.

[2] Broadhead M. L., Clark J. C., Myers D. E., Dass C. R., Choong P. F. (2011). The molecular pathogenesis of osteosarcoma: a review. *Sarcoma*.

[3] Corre I., Verrecchia F., Vincent C., Redini F., Trichet V. (2020). The osteosarcoma microenvironment: a complex but targetable ecosystem. *Cells*.

[4] Paget S. (1889). The distribution of secondary growths in cancer of the breast. *Cancer and Metastasis Reviews 1989*.

[5] Alfranca A., Martinez-Cruzado L., Tornin J. (2015). Bone microenvironment signals in osteosarcoma development. *Cellular and Molecular Life Sciences*.

[6] Vallabhaneni K. C., Hassler M. Y., Abraham A. (2016). Mesenchymalstem/stromal cells under stress increase osteosarcoma migration and apoptosis resistance via extracellular vesicle mediated communication. *PLoS One*.

[7] Pietrovito L., Leo A., Gori V. (2018). Bone marrow-derived mesenchymal stem cells promote invasiveness and transendothelialmigration of osteosarcoma cells via a mesenchymal to amoeboid transition. *Molecular Oncology*.

[8] Yoo K. H., Jang I. K., Lee M. W. (2009). Comparison of immunomodulatory properties of mesenchymal stem cells derived from adult human tissues. *Cellular Immunology*.

[9] Matsushita H., Vesely M. D., Koboldt D. C. (2012). Cancer exome analysis reveals a T-cell-dependent mechanism of cancer immunoediting. *Nature*.

[10] Mantovani A., Schioppa T., Porta C., Allavena P., Sica A. (2006). Role of tumor-associated macrophages in tumor progression and invasion. *Cancer and Metastasis Reviews*.

[11] Dwyer R. M., Potter-Beirne S. M., Harrington K. A. (2007). Monocyte chemotactic protein-1 secreted by primary breast tumors stimulates migration of mesenchymal stem cells. *Clinical Cancer Research*.

[12] Khakoo A. Y., Pati S., Anderson S. A. (2006). Human mesenchymal stem cells exert potent antitumorigenic effects in a model of Kaposi’s sarcoma. *Journal of Experimental Medicine*.

[13] Qiao L., Xu Z., Zhao T. (2008). Suppression of tumorigenesis by human mesenchymal stem cells in a hepatoma model. *Cell Research*.

[14] Kawai T., Katagiri W., Osugi M., Sugimura Y., Hibi H., Ueda M. (2015). Secretomes from bone marrow-derived mesenchymal stromal cells enhance periodontal tissue regeneration. *Cytotherapy*.

[15] Nitzsche F., Müller C., Lukomska B., Jolkkonen J., Deten A., Boltze J. (2017). Concise review: MSC adhesion cascade-insights into homing and transendothelialmigration. *Stem Cells*.

[16] Mihara K., Imai C., Coustan-Smith E. (2003). Development and functional characterization of human bone marrow mesenchymal cells immortalized by enforced expression of telomerase. *British Journal of Haematology*.

[17] Gan L., Shen H., Li X. (2021). Mesenchymal stem cells promote chemoresistance by activating autophagy in intrahepatic cholangiocarcinoma. *Oncology Reports*.

[18] Chen Y., Xu D., Yao J. (2020). Inhibition of miR-155-5p exerts anti-fibrotic effects in silicotic mice by regulating meprin alpha. *Molecular Therapy-Nucleic Acids*.

[19] Chong Z. X., Keong Yeap S., Ho W. Y. (2021). Unraveling the roles of miRNAs in regulating epithelial-to-mesenchymal transition (EMT) in osteosarcoma. *Pharmacological Research*.

[20] Cortini M., Avnet S., Baldini N. (2017). Mesenchymal stroma: role in osteosarcoma progression. *Cancer Letters*.

[21] Kaur Sarhadi V., Daddali R., Seppänen-Kaijansinkko R. (2021). Mesenchymal stem cells and extracellular vesicles in osteosarcoma pathogenesis and therapy. *International Journal of Molecular Sciences*.

[22] Namløs H. M., Kresse S. H., Müller C. R. (2012). Global gene expression profiling of human osteosarcomas reveals metastasis-associated chemokine pattern. *Sarcoma*.

[23] Sun Z., Wang S., Zhao R. C. (2014). The roles of mesenchymal stem cells in tumorinflammatory microenvironment. *Journal of Hematology & Oncology*.

[24] Lancia C., Jakob K. A., Matthew R. S. (2019). A novel method to address the association between received dose intensity and survival outcome: benefits of approaching treatment intensification at a more individualised level in a trial of the European osteosarcoma intergroup. *Cancer Chemotherapy and Pharmacology*.

[25] Ciernik I. F., Niemierko A., Harmon D. C. (2011). Proton-based radiotherapy for unresectable or incompletely resected osteosarcoma. *Cancer*.

[26] Zhao J., Dean D. C., Hornicek F. J., Yu X., Duan Z. (2020). Emerging next-generation sequencing-based discoveries for targeted osteosarcoma therapy. *Cancer Letters*.

[27] Frenette P. S., Pinho S., Lucas D., Scheiermann C. (2013). Mesenchymal stem cell: keystone of the hematopoietic stem cell niche and a stepping-stone for regenerative medicine. *Annual Review of Immunology*.

[28] Ehnman M., Chaabane W., Haglund F., Tsagkozis P. (2019). The tumor microenvironment of pediatric sarcoma: mesenchymal mechanisms regulating cell migration and metastasis. *Current Oncology Reports*.

[29] Mohseny A. B., Szuhai K., Romeo S. (2009). Osteosarcoma originates from mesenchymal stem cells in consequence of aneuploidization and genomic loss of Cdkn2. *The Journal of Pathology*.

[30] Tsukamoto S., Honoki K., Fujii H. (2012). Mesenchymal stem cells promote tumor engraftment and metastatic colonization in rat osteosarcoma model. *International Journal of Oncology*.

[31] Bonuccelli G., Avnet S., Grisendi G. (2014). Role of mesenchymal stem cells in osteosarcoma and metabolic reprogramming of tumor cells. *Oncotarget*.

[32] Le Nail L. R., Brennan M., Rosset P. (2018). Comparison of tumor- and bone marrow-derived mesenchymal stromal/stem cells from patients with high-grade osteosarcoma. *International Journal of Molecular Sciences*.

[33] Zhang J., Yu X. H., Yan Y. G., Wang C., Wang W. J. (2015). PI3K/Akt signaling in osteosarcoma. *Clinica Chimica Acta*.

[34] Engelman J. A. (2009). Targeting PI3K signalling in cancer: opportunities, challenges and limitations. *Nature Reviews Cancer*.

[35] Zhao G., Cai C., Yang T. (2013). MicroRNA-221 induces cell survival and cisplatin resistance through PI3K/Akt pathway in human osteosarcoma. *PLoS One*.

[36] Nakamizo A., Marini F., Amano T. (2005). Human bone marrow-derived mesenchymal stem cells in the treatment of gliomas. *Cancer Research*.

